# Preparation and Characterization of MWCNTs/PVDF Conductive Membrane with Cross-Linked Polymer PVA and Study on its Anti-Fouling Performance

**DOI:** 10.3390/membranes11090703

**Published:** 2021-09-14

**Authors:** Yi Ding, Zhansheng Guo, Xinan Dong, Hong You, Junxue Mei, Xuguang Hou, Zhenlin Liang, Zhipeng Li

**Affiliations:** 1 Marine College, Shandong University, Weihai 264209, China; dingyits@126.com (Y.D.); guozhansheng@sdu.edu.cn (Z.G.); meijunxue@sdu.edu.cn (J.M.); richardhoukk@163.com (X.H.); liangzhenlin@sdu.edu.cn (Z.L.); 2 State Key Laboratory of Urban Water Resources and Water Environment, School of Marine Science and Technology, Harbin Institute of Technology at Weihai, Weihai 264200, China; 13607725713@163.com (X.D.); youhong@hit.edu.cn (H.Y.)

**Keywords:** conductive membrane, carbon nanotube, cross-linked, anti-fouling

## Abstract

Based on carboxylated multi-walled carbon nanotubes (MWCNTs-COOH), a MWCNTs/PVDF conductive membrane was prepared by a vacuum filtration cross-linking method. The surface compositions and morphology of conductive membranes were studied by X-ray photoelectron spectroscopy and high-resolution field emission scanning electron microscopy, respectively. The effects of cross-linked polymeric polyvinyl alcohol (PVA) on the conductive membrane properties such as the porosity, pore size distribution, pure water flux, conductivity, hydrophilicity, stability and antifouling properties were investigated. Results showed that the addition of PVA to the MWCNTs/PVDF conductive membrane decreased the pure water flux, porosity and the conductivity. However, the hydrophilicity of the modified MWCNTs/PVDF conductive membrane was greatly improved, and the contact angle of pure water was reduced from 70.18° to 25.48° with the addition of PVA contents from 0 wt% to 0.05 wt%. Meanwhile, the conductive membranes with higher content had a relatively higher stability. It was found that the conductive functional layer of the conductive membrane had an average mass loss rate of 1.22% in the 30 min ultrasonic oscillation experiment. The tensile intensity and break elongation ratio of the conductive membrane are improved by the addition of PVA, and the durability of the conductive membrane with PVA was superior to that without PVA added. The electric assisted anti-fouling experiments of modified conductive membrane indicated that compared with the condition without electric field, the average flux attenuation of the conductive membrane was reduced by 11.2%, and the membrane flux recovery rate reached 97.05%. Moreover, the addition of PVA could accelerate the clean of the conductive membranes.

## 1. Introduction

Membrane technology is one of the most promising methods for water treatment [1,2]. In recent years, membrane-based processes have been developed and applied for different applications, including particle filtration (PF), microfiltration (MF), ultrafiltration (UF), nanofiltration (NF), reverse osmosis (RO), forward osmosis (FO), membrane distillation (MD) and membrane bioreactor (MBR) [3,4]. It is evident that remarkable progress has been made in wastewater treatment through MBR, such as wastewater reuse [5] and industrial wastewater treatment [6]. Due to increasingly stringent discharge standards, the MBR technology market is growing rapidly [7]. 

As a high-efficiency separation technology, membrane technology has an inevitable membrane fouling problem in its applications [8,9]. A variety of measures have been investigated for fouling control. Deng et al. showed membrane fouling reduction using a sponge submerged MBR [10]. Izadi et al. indicated reduced fouling with an integrated fixed bed MBR [11]. To reduce membrane fouling, the design and development of improved membranes are as important as the betterment of operational techniques. Tizchang et al. prepared silanized nanodiamond nanoparticles intercalated PSF membranes that exhibited higher flux recovery ratio (FRR) (58.93%) and improved hydrophilicity (contact angle 76.44°) [12]. Kivi et al. prepared a HDPE composite membrane for enhanced MBR performance, which exhibited higher FRR of 77.9% compared to 61.7% for the bare membrane [13]. Many studies have shown that membrane fouling can be effectively controlled through electric field force or electroflocculation [14,15]. The conductivity of the membrane was applied to prevent membrane fouling or remove foulants on the membrane during the membrane separation process [16,17,18]. 

In recent years, membrane fouling control has been achieved with other strategies such as microbial fuel cell (MFC) MBR hybrids, and the internal electric field in MFC helps to prevent substances from migrating to the surface of membrane [19]. Therefore, the cathode conductive membrane performed as both cathode and membrane in MBR/MFC coupled system. As an ideal high conductivity material, carbon nanotubes (CNTs) have been widely used in the preparation of organic/inorganic conductive separation membranes, which were endowed with better membrane properties [20,21,22]. Fan et al. used pyrolysis conversion to prepare graphene-like carbon (Cgr) as a binder to strengthen the structure of carbon nanotubes, and the CNTs/Al_2_O_3_ membrane obtained by coating interconnected CNTs on an Al_2_O_3_ substrate presented good pore-size tunability, mechanical stability, and electroconductivity [23]. In Guo et al.’ study, CNT was deposited on the surface of polyethersulfone membrane (PES), and the membrane was immersed in polypyrrole solution and then immersed in oxidant medium to produce chemical polymerization on the surface of CNT/PES membrane [24]. Lalia et al. reported that the base membrane was heat-treated to melt PVDF to provide binding sites inside the highly entangled carbon nanostructures of carbon nanotubes and enhance the mechanical strength of the membrane [25]. Due to the high degree of entanglement inside the carbon nanotubes, the dispersion of carbon nanotubes was poor and the membrane was easy to agglomerate, which led to the instability of the membrane performance [26]. However, there were also some problems such as poor impact resistance of conductive layer and poor mechanical properties of conductive membrane [27].

According to previous studies, the structure of carbon nanotubes and PVDF membranes was mainly maintained by weak van der Waals force, which was easy to be destroyed in water flow [28,29]. Therefore, enhancing the stability of CNTs conductive layer was an important key point in the preparation of conductive membrane. It was reported that polyvinyl alcohol (PVA) and CNTs can be connected together by covalent bonds between hydroxyl groups on PVA molecules and carboxyl groups on carboxylated multi-walled carbon nanotubes (MWCNTs) [30,31].

Therefore, in this study, MWCNTs/PVDF conductive membranes were prepared by adjusting the concentration of cross-linked polymer PVA. The structure, conductivity, hydrophilicity and stability of the conductive membrane were investigated with the addition of PVA, and the performance of the conductive membrane combined with the electric field to filter the yeast suspension was explored through the electric-assisted filtration experiment. The results showed that with the addition of PVA, MWCNTs/PVDF conductive membrane had strong stability, excellent hydrophilicity and anti-fouling properties. The conductive membrane studied in this paper would have good economy and good application prospect in MFC-MBR system, and further improve the understanding of conductive membrane fouling behaviors in MBR for wastewater treatment.

## 2. Materials and Methods

### 2.1. Preparation of MWCNTs/PVDF Conductive Membrane

In this experiment, MWCNTs/PVDF conductive membrane was prepared by a vacuum filtration crosslinking method [32]. The preparation process of MWCNTs/PVDF conductive composite membrane mainly included two steps: first, the carboxylated multi-walled carbon nanotubes solution was mixed with polyvinyl alcohol solution, and the mixed solution was filtered to PVDF membrane using vacuum filtration; Second, the membrane was immersed in glutaraldehyde solution for crosslinking under the conditions of heating in the presence of hydrochloric acid. Firstly, the commercial PVDF membrane was pretreated by ultrasound with ethanol at 50 W for 15 min. After washing the surface with deionized water, the membrane was dried in an oven at 50 °C for 24 h. In order to improve the dispersion of CNTs, a certain amount of sodium dodecylbenzene sulfonate (SDBS) was added, and the ratio of MWCNTs-COOH: SDBS was 1: 10; Then, the carbon nanotube solution was pretreated with ultrasonic wave at 400 W and 18 °C for, and the ultrasonic wave was set to stop for 4 s every 2 s to prevent the solution from foaming.

Different amounts of PVA were weighed and dissolved in deionized water at 95 °C for 1 hour to prepare PVA solutions with concentrations of 0.01 wt%, 0.02 wt%, 0.03 wt%, 0.04 wt% and 0.05 wt%. The pretreated PVDF flat membrane was put into a vacuum filter device, and a certain amount of carbon nanotube solution and PVA solution were mixed and then poured into the vacuum filtration device for suction filtration. The membrane prepared by suction filtration was immersed in the crosslinking solution composed of 0.01 mol/L glutaraldehyde and 0.01 mol/L hydrochloric acid and heated at 90 °C for 5 h. Then the membrane was taken out from the heating bath. After cooling to room temperature, the residual solution on the membrane surface was cleaned with deionized water and dried at 50 °C for 24 h.

### 2.2. Characterization of MWCNTs/PVDF Conductive Membrane

#### 2.2.1. XPS Study

X-ray photoelectron spectroscopy (Escalab 250xi, Thermo Fisher Scientific, Waltham, MA, USA) was used to quantitatively analyze the elements and composition of the conductive layer on the surface of the conductive membrane.

#### 2.2.2. Morphology Study of Membranes

The surface morphology of MWCNTs/PVDF conductive membrane was observed by high resolution field emission scanning electron microscope (FESEM) (Merlin Compact, Zeiss, Jena, Germany).

#### 2.2.3. Porosity

Membrane porosity was measured according to its dry-wet weight. The membrane kept in distilled water was weighed after wiping off superficial water. Then the wet membrane was placed in an air-circulating oven at 60 °C for 24 h and then further dried in a vacuum oven at 80 °C for 24 h before measuring the dry mass. According to the wet sample mass and the dry sample mass, the porosity of membrane was calculated.

#### 2.2.4. Pore Size Distribution

The membrane pore size was determined by bubble pressure membrane pore size analyzer (BSD-PB, Beishide Instrument Technology Co., Ltd., Beijing, China).

#### 2.2.5. Pure Water Flux

A cross flow filtration experimental set-up was used to measure the pure water flux of the prepared membranes. The set-up was fed with distilled water at a transmembrane pressure of 0.01 MPa after the membranes were pre-pressurized for 50 min at 0.02 MPa.

#### 2.2.6. Conductivity

Four probe resistance meter (RTS-4, Four probe technology Co., Ltd., Guangzhou, China) was used to measure the conductivity of the conductive membrane. Before the measurement, the membrane was dried at 50 °C for 24 h. Five evenly distributed points on the surface of the membrane were selected for test and the average value was calculated.

#### 2.2.7. Contact Angle

The water contact angle of conductive membrane was measured by optical contact angle meter (SL200KS, Kono Industries Co., Ltd., Boston, MA, USA).

### 2.3. Stability of Conductive Membrane

#### 2.3.1. Mass Loss Rate

The effect of polyvinyl alcohol concentration on the stability of conductive functional layer was studied by ultrasonic oscillation experiment using ultrasonic cleaner (SK5210LHC, Kedao Ultrasonic Instrument Co., Ltd., Shanghai, China). The mechanical stability of the conductive layer was characterized by calculating the mass loss of the conductive layer in the 30 min ultrasonic oscillation experiment. Before weighing, the conductive membrane would be strictly sealed and dried in an oven at 55 °C for 24 h to remove moisture and ensure that there were no pollutants on the surface.

The mass loss rate of conductive layer was calculated as follows:

(1) $$Mass\;loss\;rate=\frac{M_{1}-M_{2}}{M_{3}-M_{4}}\times 100\%$$

where: *M*_1_ is dry weight of the conductive membrane before ultrasonic oscillation (g), *M*_2_ is dry weight of the conductive membrane after ultrasonic oscillation (g), *M*_3_ is the dry weight of MWCNTs/PVDF conductive membrane (g) and *M*_4_ is the dry weight of the PVDF base membrane (g).

#### 2.3.2. Mechanical Property Test

The tensile intensity and break elongation ratio of the membrane were determined by a universal electronic strength measurement (LLY-06, Electronic Instrument Co., Ltd, Laizhou, China).

#### 2.3.3. Durability

Bovine serum albumin (BSA) is one of the most widely studied proteins. Many studies have used BSA as model protein for systematically studying the fouling behavior of membranes. To evaluate the durability of the conductive membrane, a ten-cycle BSA rejection experiment was carried out. The BSA concentrations in permeate and feed were determined by an UV-spectrophotometer (UV–2450, Shimadzu, Kyoto, Japan). BSA rejection (*R*) was calculated according to Equation (2):

(2) $$R=1-\frac{C_{p}}{C_{f}}$$

where *C_p_* and *C_f_* are the concentrations of protein in permeate and initial feed, respectively.

### 2.4. Membrane Fouling Studies of Conductive Membrane

**Figure 1:**
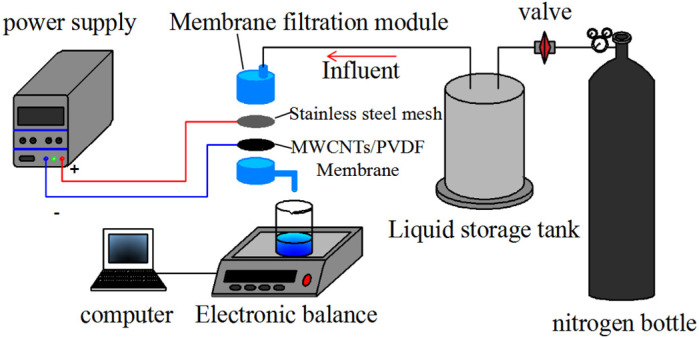
Applied external electric field membrane filtering device.

A self-made membrane filtration reactor was used to test the anti-fouling performance of conductive membrane. The external electric field membrane filtration device was shown in Figure 1, and in the membrane filter module, the anode was on the top and the cathode was on the bottom. The anode was stainless steel mesh and the cathode was MWCNTs/PVDF conductive membrane.

In the anti-fouling experiment, 2 g/L yeast suspension was used as the target solution and the pressure was 0.01 MPa. During the experiment, a DC regulated power supply (KXN-645D, Zhaoxin Electronic Instrument Equipment Co., Ltd., Shenzhen, China) was used to apply a certain intensity of micro electric field between the anode and cathode. By comparing the experimental data with and without external electric field, the effect of external electric field on reducing membrane fouling was analyzed. By measuring the flux of fouling substances and the flux after physical cleaning, the flux change and flux recovery rate of conductive membrane were calculated to further investigate its anti-fouling performance.

The membrane flux was calculated as follows:

(3) $$J=\frac{V\times 60}{A\times t\times p\times 1000}$$

where: *J* is the permeation flux(L/m^2^·h·bar), *V* is the volume of collected permeate (mL), *T* is the permeation time (min), *A* is the effective membrane filtration area (m^2^) and *P* is the osmotic pressure (bar).

### 2.5. Membrane Cleaning of Conductive Membrane

The membranes fouled by BSA solution were soaked in NaClO aqueous solution (0.5 L NaClO + 10 L deionized water) for 10 min. Then they were rinsed by distilled water and the fluxes (J_hw_) were measured. The ratio (J_hw_/J_w_) was calculated. J_w_ is the clean membrane water flux at steady state.

### 2.6. Statistics and Analysis

In this study, the membrane properties of conductive membranes were characterized and tested by three groups of parallel experiments. The statistical analysis of the data was made to ensure the data validity, and the average value of the results were reported.

## 3. Results

### 3.1. Effect of Added PVA on Surface Compositions and Morphology of Conductive Membrane

#### 3.1.1. Surface Compositions of Conductive Membrane

**Figure 2:**
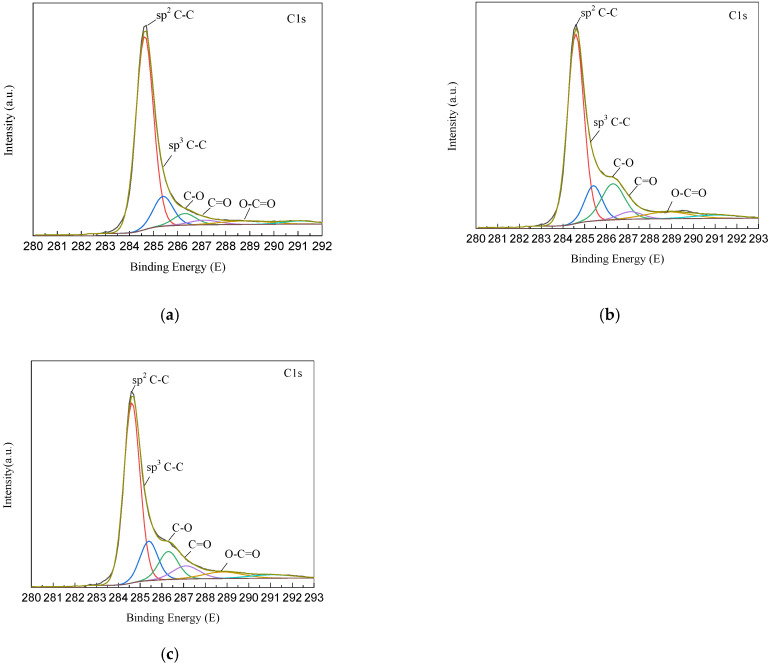
XPS image of MWCNTs/PVDF conductive membrane: (**a**) Conductive membrane without PVA; (**b**) Conductive membrane with PVA added; (**c**) Conductive membrane crosslinked with GA.

In order to investigate the effect of added PVA on the surface compositions of MWCNTs/PVDF conductive membrane, different types of MWCNTs/PVDF were analyzed by X-ray photoelectron spectroscopy. The XPS spectra of MWCNTs/PVDF conductive membrane at different preparation stages were shown in Figure 2. According to the results of C1s peaks in Figure 2, the carbon element in the conductive membrane mainly existed in the form of sp^2^C-C(284.6 eV), sp^3^C-C(285.4 eV), C-O(286.3 eV), C=O(287.1 eV) and O-C=O(288.8 eV). The results showed that the C-O bond content of the membrane increased from 5.51% to 16.18% compared with that of the membrane without PVA, which indicated that the crosslinked polymer PVA was effectively entangled on the carbon nanotubes and formed a conductive functional layer with vacuum filtration. Polyvinyl alcohol contained a large number of C-O groups that have not participated in the reaction, so it increased the content of C-O groups in the conductive layer of carbon nanotubes. Compared with Figure 2b,c, it can be found that the content of C-O bond decreased from 16.18% to 11.09%, which indicated that PVA and CNTs were interconnected to form a network structure under the action of glutaraldehyde.

#### 3.1.2. Morphology Analysis of Conductive Membrane

**Figure 3:**
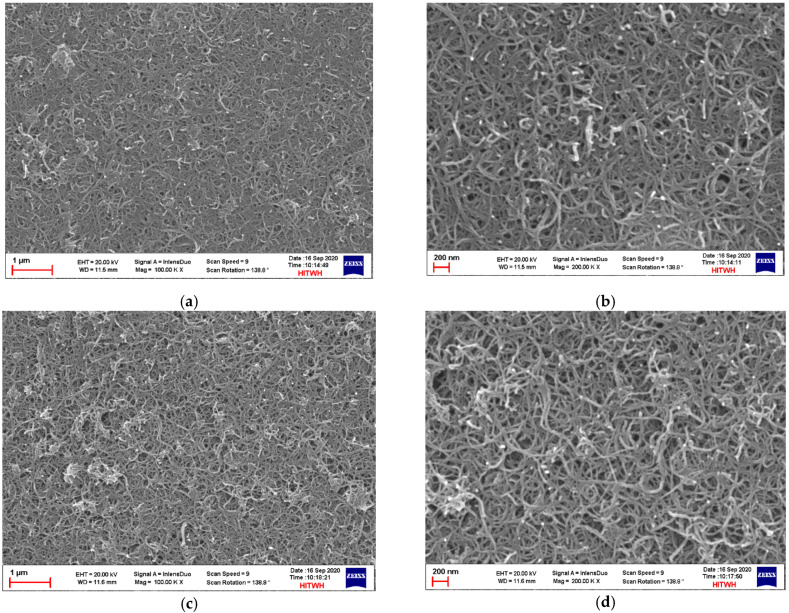
FESEM images of MWCNTs/PVDF conductive membrane: Surface morphology of MWCNTs/PVDF conductive composite membrane before crosslinking with 1 μm (**a**) and 200 nm (**b**); Surface morphology of crosslinked MWCNTs/PVDF conductive composite membrane with 1 μm (**c**) and 200 nm (**d**).

FESEM analysis is an important technique to study the membrane morphology and qualitative information of surface morphology of the membranes. FESEM images of MWCNTs/PVDF conductive composite membrane at different preparation stages are shown in Figure 3. During the crosslinking reaction process, the crosslinking polymer polyvinyl alcohol and crosslinking agent glutaraldehyde on the surface of the conductive membrane could join the reaction and fully contact with the multi-walled carbon nanotubes. By comparing the surface FESEM image of the conductive membrane before and after crosslinking, it was found that the overall roughness of the conductive functional layer has increased after crosslinking reaction, and the number of interconnecting holes between multi-walled carbon nanotubes also increased and the size of interconnecting holes became larger. These crosslinking substances were also used to connect the multi-walled carbon nanotubes and help to strengthen the overall bonding strength of the conductive functional layer.

### 3.2. Effect of Added PVA Contents on Porosity of Conductive Membrane

**Figure 4:**
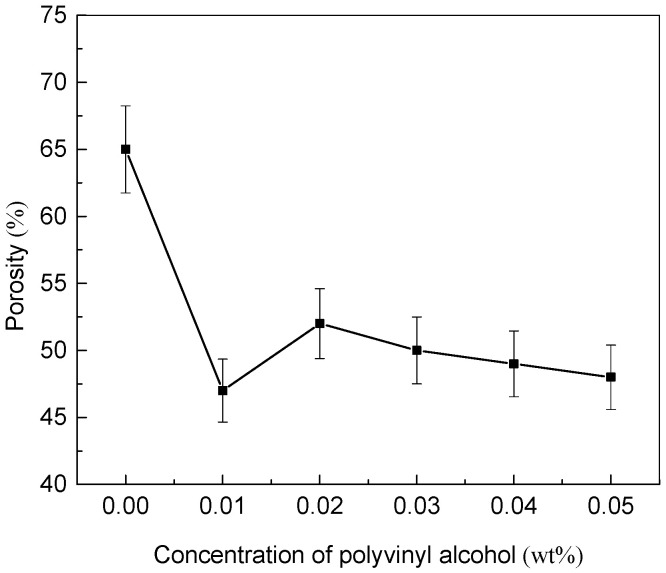
Effect of added PVA contents on the porosity of MWCNTs/PVDF conductive membranes.

The porosity is one of the important parameters to determine the performance of membranes. The porosity of MWCNTs/PVDF conductive membranes with different PVA contents was shown in Figure 4. When the concentration of PVA increased from 0 wt% to 0.01 wt%, the porosity of the conductive membrane decreased significantly. It was found that with the PVA concentration of 0.01 wt%, a small amount of agglomeration of carbon nanotubes appeared when the PVA was mixed with the carbon nanotube solution. Combined with the analysis of relevant research, polyvinyl alcohol would be entangled on carbon nanotubes, increasing the hydrophilicity of the carbon nanotubes to make the dispersion stability better, and the hydroxyl groups of different complexes would be easy to agglomerate due to strong hydrogen bonding [33,34,35]. Therefore, it was speculated that when the concentration of PVA was too low, the stronger hydrogen bonding led to the agglomeration of carboxylated multi-walled carbon nanotubes. The agglomerations blocked the pores or caves of conductive membranes and led to the decrease of porosity. When the concentration of PVA was 0.02 wt%, the porosity of conductive membrane increased. When the concentration of PVA was between 0.02 wt% to 0.05 wt%, the porosity of conductive membrane decreased with the increase of PVA content.

### 3.3. Effect of Added PVA Contents on Pore Size and Permeability

**Figure 5:**
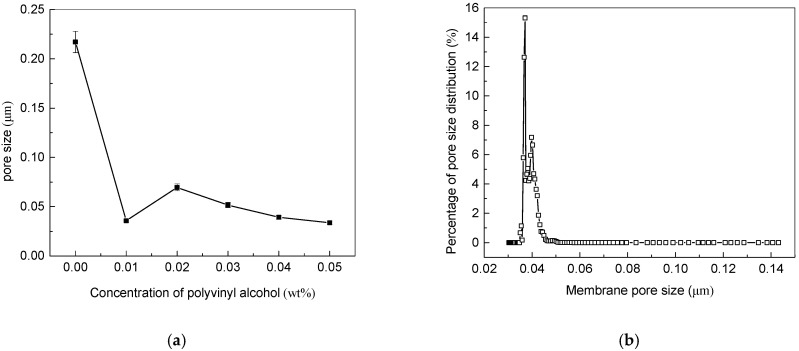
Pore size analysis of MWCNTs/PVDF conductive membrane: (**a**) Change of average pore diameter of conductive membrane; (**b**) Pore size distribution of conductive membrane (PVA concentration was 0.05 wt%).

The bubble point method was used to determine the pore size of the membrane, and the results were shown in Figure 5. The average pore diameter of the original PVDF base membrane was 4 μm. It can be seen from Figure 5a that the average pore diameter of the conductive membrane was 0.22 μm after adding carbon nanotubes which was due to the entanglement of carbon nanotubes on the surface of PVDF membrane. According to the characteristics of the preparation process, after the addition of cross-linked polymer polyvinyl alcohol, the pore size of MWCNTs/PVDF conductive membrane would be further reduced after the cross-linking reaction.

When the concentration of polyvinyl alcohol increased from 0 wt% to 0.01 wt%, the average pore diameter of the conductive membrane decreased from 0.22 μm down to 0.036 μm. The reason was that with the polyvinyl alcohol concentration of 0.01 wt%, a small amount of agglomeration of carbon nanotubes appeared when it was mixed with carbon nanotube solution. When the concentration was between 0.02 wt% to 0.05 wt%, the pore size of conductive membrane decreased with the increase of polyvinyl alcohol content.

The pore size distribution of the conductive membrane with the PVA concentration of 0.05 wt% was shown in Figure 5b. It could be seen that the pore size distribution of the membrane was relatively concentrated, and the pore diameter of conductive membrane was concentrated in 0.035–0.045 μm. At the same time, it also showed that the carboxylated multi-walled carbon nanotubes were dispersed evenly in the solution by adding surfactants and high-power and long-time ultrasound, which effectively improved the dispersion of carbon nanotubes.

**Figure 6:**
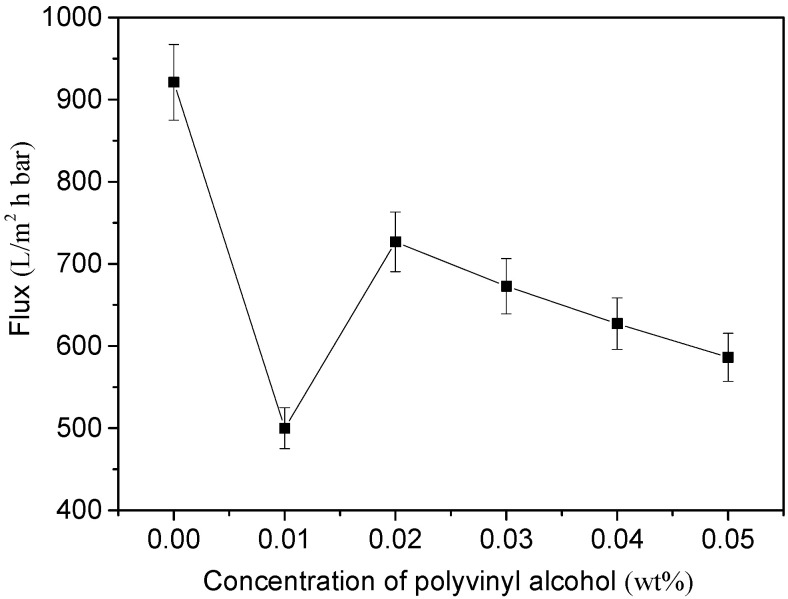
Effect of added PVA contents on pure water flux of MWCNTs/PVDF conductive membranes.

The pure water flux of MWCNTs/PVDF conductive membrane was tested, and the result is shown in Figure 6. The pure water flux of MWCNTs/PVDF conductive membrane was measured in three groups of parallel experiments. It can be seen from the figure that by comparing the pure water flux between the conductive membranes with different polyvinyl alcohol concentrations, it was found that the conductive membrane flux is the lowest when the polyvinyl alcohol concentration was 0.01 wt%. Overall, the pure water flux of the conductive membrane with different PVA concentrations was little different. Affected by the load of cross-linked polymer, the change trend of the pure water flux of the conductive membrane was consistent with that of the membrane pore size in Figure 5.

### 3.4. Effect of Added PVA Contents on Conductivity

Conductivity is one of the important properties of conductive membrane. Carbon nanotubes provided better conductivity for conductive membrane because of their excellent conductivity [36,37].

**Figure 7:**
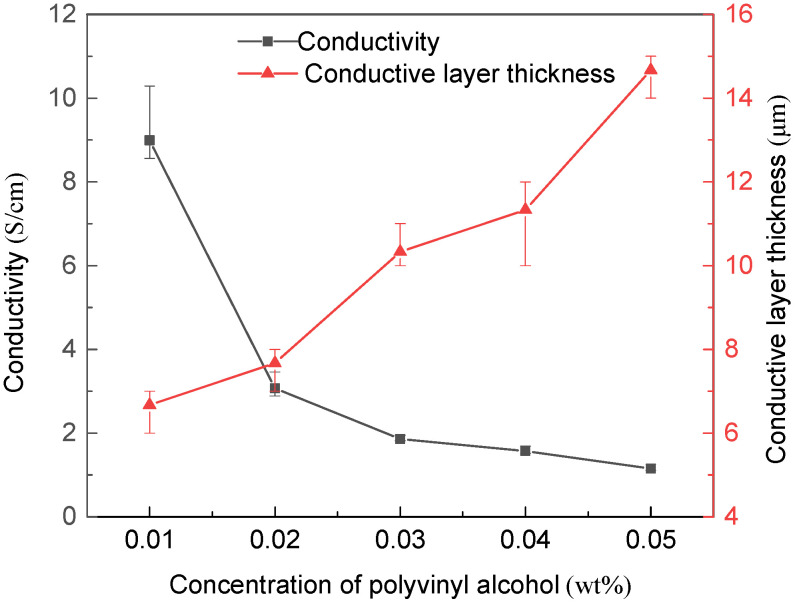
Change of conductivity and conductive layer thickness of MWCNTs/PVDF conductive membrane.

The changes of conductivity and conductive layer thickness of MWCNTs/PVDF conductive membrane were shown in Figure 7. It can be seen that with the addition of polyvinyl alcohol during the preparation process, the PVA was bound between carbon nanotubes. When the polyvinyl alcohol concentration gradually increased from 0.01 wt% to 0.05 wt%, the binding strength and number increased, and the average thickness of the conductive layer of the conductive membrane increased from 6.67 μm to 14.67 μm. Under the interference of polyvinyl alcohol insulation, the electron transfer resistance increased, so the average conductivity of the functional layer decreased from 8.99 S/cm to 1.15 S/cm with the polyvinyl alcohol concentration gradually increasing from 0.01 wt% to 0.05 wt%. The test results were similar to the previous experimental results [38]. The stability of the conductive membrane increased with sacrificing part of the conductivity. When the polyvinyl alcohol concentration gradually increased from 0.01 wt% to 0.05 wt%, the MWCNTs/PVDF conductive membrane exhibited relatively good electrical conductivity would serve as cathode in MFC/MBR system to improve the removal efficiency and alleviate membrane fouling. In MBR, the surfaces of sludge flocs were negatively charged. If a negative voltage was applied on the side passing through the membrane, the moving rate of sludge flocs to the membrane surface under the repulsion of electric field force would be greatly reduced, which reduced the attachment rate of foulants on the membrane surface, thus mitigating the membrane fouling. In future research, the MWCNTs/PVDF conductive membrane with relatively good electrical conductivity served as cathode in MFC/MBR system would improve the removal efficiency and alleviate membrane fouling. 

### 3.5. Effect of Added PVA Contents on Hydrophilicity

The hydrophilicity of the membrane directly affected its anti-fouling performance in practical application [39,40]. Therefore, carboxylated multiwall carbon nanotubes were used to prepare conductive membrane in this experiment. Compared with conventional carbon nanotubes, carboxylated carbon nanotubes can greatly improve the hydrophilicity of carbon nanotubes due to the existence of hydrophilic carboxyl groups [41]. At the same time, the hydrophilic properties of the membrane were further enhanced by the addition of hydrophilic cross-linked polymer polyvinyl alcohol.

**Figure 8:**
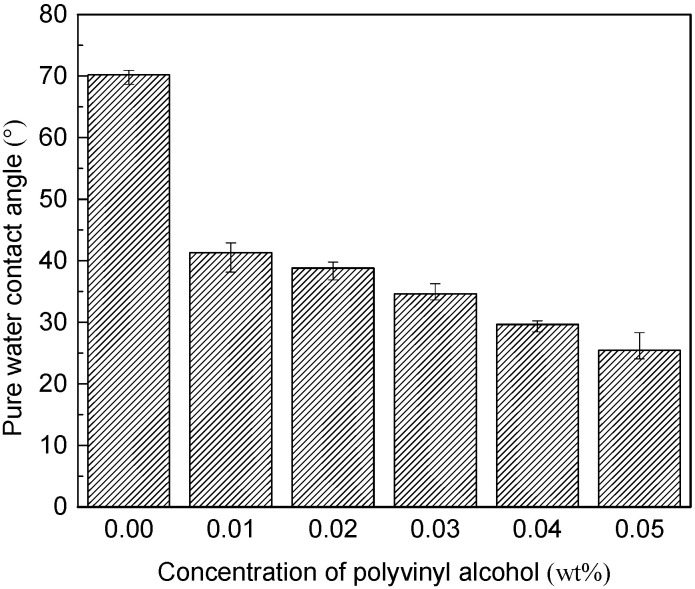
Pure water contact angle of MWCNTs/PVDF conductive membrane.

The pure water contact angle of MWCNTs/PVDF conductive membrane at different concentrations was shown in Figure 8. The pure water contact angle of the original PVDF membrane was 87.14°, and the hydrophilicity of the conductive membrane loaded with carboxylated multiwall carbon nanotubes was improved to a certain extent, and the pure water contact angle was reduced to 70.18°.

It can be seen from the figure that after the addition of cross-linked polymer polyvinyl alcohol, the hydrophilicity of the conductive membrane was greatly improved, and the pure water contact angle gradually decreased with the increased of polyvinyl alcohol concentration. When the concentration of polyvinyl alcohol increased to 0.05 wt%, the pure water contact angle of the conductive membrane decreased to 25.48°. It had been reported that high fouling rates was attributed to high hydrophobic foulants in MBR [42]. The hydrophobic membrane foulants such as proteins, humic-like and fulvic-like materials, showed stronger interaction with hydrophobic membranes. The hydrophilicity of the membrane was greatly improved compared with that before modification, which was also helpful to further optimize the anti-fouling performance of the conductive membrane.

### 3.6. Stability Analysis of Conductive Membrane

#### 3.6.1. Mass Loss Rate of Conductive Membrane

In this study, the covalent bond between the hydroxyl group on the polyvinyl alcohol molecule and the carboxyl group on the carboxylated carbon nanotubes was used to connect them together, and the cross-linking reaction greatly increased the mechanical strength of the membrane. The effect of polyvinyl alcohol concentration of crosslinked polymer on the stability of conductive functional layer was explored through ultrasonic oscillation experiment. The mass loss of conductive layer in 30 min ultrasonic oscillation experiment was calculated to characterize the mechanical stability of conductive layer. 

**Figure 9:**
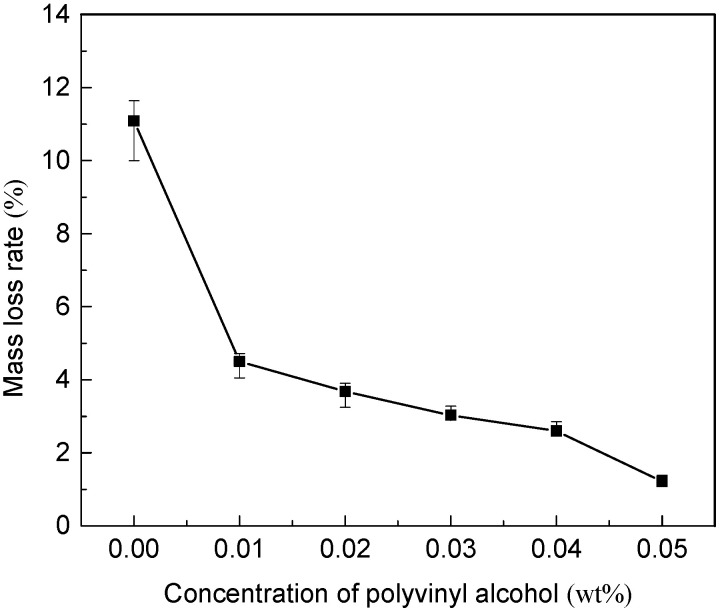
Changes in the mass loss rate of the conductive layer of the MWCNTs/PVDF conductive membrane.

The change in the mass loss rate of the conductive layer of the MWCNTs/PVDF conductive membrane was shown in Figure 9. When the loading amount of carboxylated MWCNTs was 0.5 mg/cm^2^, the average mass loss rate of MWCNTs/PVDF conductive membrane without cross-linked polymer polyvinyl alcohol was 11.09%. The mass loss rate of the conductive layer of the conductive membrane decreased by 59.42% after the addition of cross-linked polymer polyvinyl alcohol, indicating that the addition of polyvinyl alcohol effectively enhanced the physical stability of the conductive layer. With the polyvinyl alcohol concentration of 0.05 wt%, the average mass loss rate of the conductive layer was only 1.22%, and the mass loss was as low as 10^−4^ g, indicating that the stability of the conductive layer was excellent.

#### 3.6.2. Mechanical Property Tests of the Conductive Membrane

The tensile intensity and break elongation ratio of the MWCNTs/PVDF conductive membrane without and with the PVA are listed in Table 1. Both mechanical properties are improved by the addition of PVA, especially the tensile strength.

#### 3.6.3. Durability of Conductive Membrane

**Figure 10:**
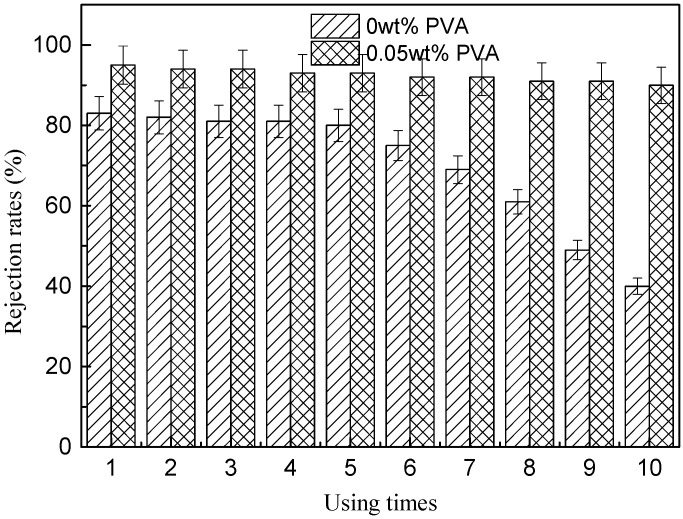
The durability of the MWCNTs/PVDF conductive membrane without and with PVA.

To evaluate the durability of the MWCNTs/PVDF conductive membrane, a ten-cycle BSA rejection experiment was carried out for MWCNTs/PVDF conductive membrane without and with the PVA concentration of 0.05 wt%, as shown in Figure 10. After 120 min of BSA rejection experiment, the MWCNTs/PVDF conductive membrane was cleaned and transferred to a filtration apparatus to carry out the subsequent BSA rejection experiment. The rejection efficiency of the MWCNTs/PVDF conductive membrane without PVA added began to decrease after five cycles. The BSA rejection rates was decreased to 80% after the first five cycles and decreased to 69% after seven cycles, 61% after eight cycles, 49% after nine cycles and 40% after ten cycles. The rejection efficiency of the MWCNTs/PVDF conductive membrane with PVA added was more than 90% after ten cycles. It was indicated that the durability of the MWCNTs/PVDF conductive membrane with PVA was superior to that without added PVA.

### 3.7. Anti-Fouling Properties of the Conductive Membrane

#### 3.7.1. Fouling Studies of the Conductive Membrane

Before the experiment, a nanoparticle size and zeta potential analyzer was used to analyze the prepared yeast suspension. The average particle size of the prepared yeast suspension was 4554 nm, and the zeta potential was –0.527 mv. MWCNTs/PVDF conductive membrane with polyvinyl alcohol concentration of 0.05 wt% was used for three cycles of filtration experiment at 0.01 Mpa. The time of each filtration cycle was 120 min. After each cycle of operation, the conductive membrane was physically cleaned and placed into the reactor for the next cycle of filtration experiment.

**Figure 11:**
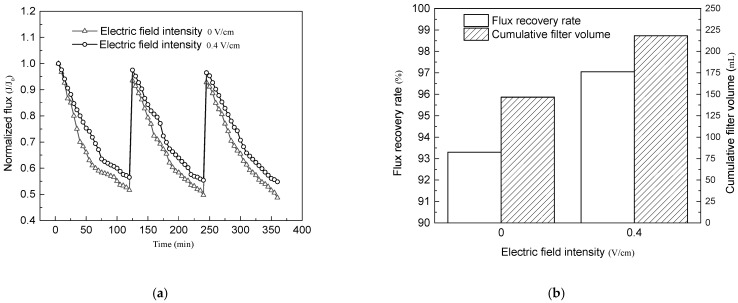
Filtration performance of MWCNTs/PVDF conductive membrane for yeast suspension: (**a**) Normalized flux varies with filtration time; (**b**) Flux recovery rate and cumulative filtered solution volume under different conditions.

The filtration performance of MWCNTs/PVDF conductive membrane for yeast suspension is shown in Figure 11. It can be seen from Figure 11a that the decay rate of membrane flux became slower when negative bias was applied to the cathode than that with no external electric field applied. When the intensity of external electric field was 0.4 V/cm, the membrane flux loss decreased by 9.5% after 120 min, 11.5% after 240 min and 12.6% after 360 min, indicating that the membrane fouling of conductive membrane was improved when the external electric field was applied. The membrane fouling tendency was slower than that under ordinary conditions with the increase of filtration time. 

The comparison of conductivity membrane flux recovery rate and cumulative filtered solution volume under two kinds of filtration conditions was shown in Figure 11b. In this paper, the cumulative filtration volume is used to represent the overall flux of the membrane. It can be seen from the Figure 11b that when no external electric field was applied, the cumulative filtration volume of the conductive membrane for yeast suspension was 146.57 mL. When a 0.4 V/cm external electric field was applied, the cumulative filtration volume was 218.10 mL. It could be seen that under the condition of 0.4 V/cm enhancement of external electric field, the flux of conductive membrane was increased by 48.8% compared with that without external electric field, and the flux recovery rate of conductive membrane was as high as 97%.

The reason for this phenomenon was that with the solution filtering, the conductive membrane surface was electronegative under the action of electric field, while the zeta potential of yeast suspension was negative. Therefore, negatively charged yeast particles were not easy to adhere to the surface of conductive membrane under the electrostatic repulsion between the same electrical properties.

#### 3.7.2. Conductive Membrane Cleaning Studies

**Figure 12:**
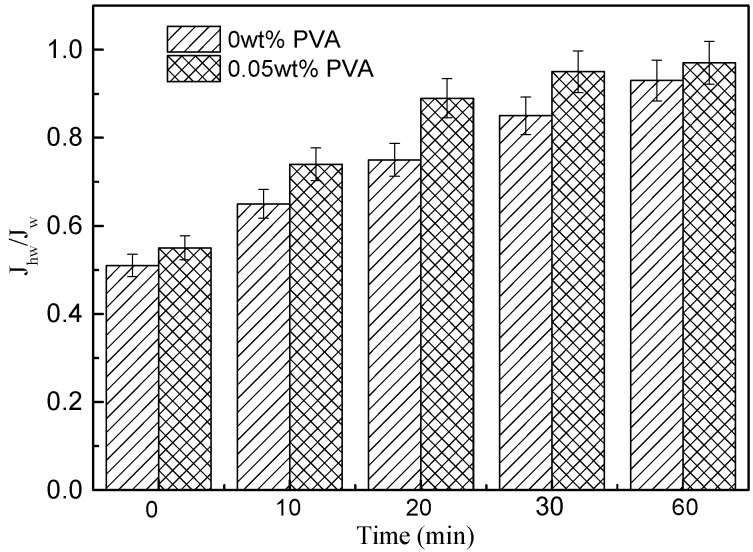
Effect of operation time on J_hw_/J_w_ of PVA 0 wt% and PVA 0.05 wt% conductive membrane.

The fouled conductive membranes by BSA were washed by NaClO aqueous solution (0.5 L NaClO + 10 L deionized water) for 10 min. The effect of operation time on J_hw_/J_w_ value of MWCNTs/PVDF conductive membrane without and with the PVA concentration of 0.05wt% is shown in Figure 12. The J_hw_/J_w_ value of conductive membrane without PVA increased slowly while J_hw_/J_w_ value of conductive membrane with PVA increased quickly with the increase of operating time by NaClO aqueous solution. After 20 min run, the J_hw_/J_w_ value of conductive membrane with PVA attained nearly 90% while that of conductive membrane without PVA was lower than 80%. It meant that the addition of PVA could accelerate the clean of the conductive membranes.

## 4. Conclusions

In this study, MWCNTs/PVDF conductive membranes were prepared by a vacuum filtration crosslinking method. The addition of crosslinked polymers PVA had a strong effect on the properties of the MWCNTs/PVDF conductive membrane. The porosity, pore size distribution, pure water flux and conductivity of the conductive membrane were inversely proportional to the concentration of cross-linked polymer PVA, and the hydrophilicity, stability and anti-fouling of conductive membrane were directly proportional to that factor. With the addition of 0.05 wt%, PVA the average porosity of the membrane was 48%, and the average conductivity of the membrane was 1.154 S/cm, and the average pore diameter of the membrane was 33.7 nm. The pure water contact angle of the modified conductive membrane decreased to 25.477°, and the hydrophilicity of the membrane was greatly improved. The conductive membranes with higher PVA content had a relatively higher stability. The conductive functional layer remained intact during 30 min ultrasonic oscillation test, with an average mass loss rate of 1.22%. The break elongation ratio and tensile intensity were 5.9% and 14.4 N, respectively. Under the condition of electrical assistance, the MWCNTs/PVDF conductive membrane exhibited excellent anti-fouling performance. When the intensity of external electric field was 0.4 V/cm, the attenuation of membrane flux was reduced by 11.2% compared with that without an external electric field. It exhibited an excellent cleaning effect when crosslinked polymeric PVA was introduced into the conductive membranes. In future research, the conductive membrane studied in this paper should have good economy and good application prospects in MFC-MBR systems.

## Figures and Tables

**Table 1:** Mechanical properties of membrane without and with the PVA.

Item	MWCNTs/PVDF Conductive Membrane (0 wt% PVA)	MWCNTs/PVDF Conductive Membrane with PVA (0.05 wt% PVA)
Break elongation ratio (%)	4.1	5.9
Tensile intensity (N)	11.5	14.4

## References

[1] Ding Y., Guo Z., Mei J., Liang Z., Li Z., Hou X. (2020). Investigation into the Novel Microalgae Membrane Bioreactor with Internal Circulating Fluidized Bed for Marine Aquaculture Wastewater Treatment. Membranes.

[2] Skoczko I., Puzowski P., Szatyłowicz E. (2020). Experience from the Implementation and Operation of the Biological Membrane Reactor (MBR) at the Modernized Wastewater Treatment Plant in Wydminy. Water.

[3] Lee A., Elam J.W., Darling S.B. (2016). Membrane materials for water purification: Design, development, and application. Environ. Sci. Water Res. Technol..

[4] Piekutin J., Skoczko I. (2014). Use of stripping tower and reverse osmosis in removal of petroleum hydrocarbons from water. Desalination Water Treat..

[5] Falizi N.J., Hacıfazlıoğlu M.C., Parlar İ., Kabay N., Pek T.Ö., Yüksel M. (2018). Evaluation of MBR treated industrial wastewater quality before and after desalination by NF and RO processes for agricultural reuse. J. Water Process. Eng..

[6] Mutamim N.S.A., Noor Z.Z., Hassan M.A.A., Olsson G. (2012). Application of membrane bioreactor technology in treating high strength industrial wastewater: A performance review. Desalination.

[7] Pervez M.N., Balakrishnan M., Hasan S.W., Choo K.H., Zhao Y., Cai Y., Zarra T., Belgiorno V., Naddeo V. (2020). A critical review on nanomaterials membrane bioreactor (NMs-MBR) for wastewater treatment. npj Clean Water.

[8] Guo W., Ngo H.-H., Li J. (2012). A mini-review on membrane fouling. Bioresour. Technol..

[9] Tijing L.D., Woo Y.C., Choi J.-S., Lee S., Kim S.-H., Shon H.K. (2015). Fouling and its control in membrane distillation—A review. J. Membr. Sci..

[10] Deng L., Guo W., Ngo H.H., Zhang J., Liang S., Xia S., Zhang Z., Li J. (2014). A comparison study on membrane fouling in a sponge-submerged membrane bioreactor and a conventional membrane bioreactor—ScienceDirect. Bioresour. Technol..

[11] Izadi A., Hosseini M., Darzi G.N., Bidhendi G.N., Shariati F.P. (2019). Performance of an integrated Fixed Bed Membrane Bioreactor (FBMBR) applied to pollutant removal from paper-recycling wastewater. Water Resour. Ind..

[12] Tizchang A., Jafarzadeh Y., Yegani R., Khakpour S. (2019). The effects of pristine and silanized nanodiamond on the performance of polysulfone membranes for wastewater treatment by MBR system. J. Environ. Chem. Eng..

[13] Kivi M.A., Alinia H., Jafarzadeh Y., Yegani R. (2019). High-density polyethylene membranes embedded with carboxylated and polyethylene glycol-grafted nanodiamond to be used in membrane bioreactors. J. Appl. Polym..

[14] Du Z., Ji M., Li R. (2021). Alleviation of membrane fouling and enhancement of trace organic compounds removal in an electric field assisted microfiltration system. Chem. Eng. J..

[15] Dong Z., Shang W., Dong W., Zhao L., Li M., Wang R. (2018). Suppression of membrane fouling in the ceramic membrane bioreactor (CMBR) by minute electric field. Bioresour. Technol..

[16] Pan Z., Song C., Li L., Wang H., Pan Y., Wang C., Sun F. (2019). Membrane technology coupled with electrochemical advanced oxidation processes for organic wastewater treatment: Recent advances and future prospects. Chem. Eng. J..

[17] Liu L., Liu J., Bo G., Yang F., Crittenden J., Chen Y. (2013). Conductive and hydrophilic polypyrrole modified membrane cathodes and fouling reduction in MBR. J. Membr. Sci..

[18] Li P., Yang C., Sun F., Li X.-Y. (2021). Fabrication of conductive ceramic membranes for electrically assisted fouling control during membrane filtration for wastewater treatment. Chemosphere.

[19] Li Y., Cheng C., Bai S., Jing L., Zhao Z., Liu L. (2019). The performance of Pd-rGO electro-deposited PVDF/carbon fiber cloth composite membrane in MBR/MFC coupled system. Chem. Eng. J..

[20] Khezraqa H., Etemadi H., Qazvini H., Salami-Kalajahi M. (2021). Novel polycarbonate membrane embedded with multi-walled carbon nanotube for water treatment: A comparative study between bovine serum albumin and humic acid removal. Polym. Bull..

[21] Xu X., Zhang H., Yu M., Wang Y., Gao T., Yang F. (2019). Conductive thin film nanocomposite forward osmosis membrane (TFN-FO) blended with carbon nanoparticles for membrane fouling control. Sci. Total. Environ..

[22] Dhand V., Hong S.K., Li L., Kim J.-M., Kim S.H., Rhee K.Y., Lee H.W. (2019). Fabrication of robust, ultrathin and light weight, hydrophilic, PVDF-CNT membrane composite for salt rejection. Compos. Part B Eng..

[23] Fan X., Zhao H., Liu Y., Quan X., Yu H., Chen S. (2015). Enhanced permeability, selectivity, and antifouling ability of CNTs/Al2O3 membrane under electrochemical assistance. Environ. Sci. Technol..

[24] Guo Z.Y., Yuan X.S., Geng H.Z., Wang L.D., Jing L.C., Gu Z.Z. (2018). High conductive PPy–CNT surface-modified PES membrane with anti-fouling property. Appl. Nanosci..

[25] Lalia B.S., Ahmed F.E., Shah T., Hilal N., Hashaikeh R. (2015). Electrically conductive membranes based on carbon nanostructures for self-cleaning of biofouling. Desalination.

[26] Jorio A., Dresselhaus G., Dresselhaus M.S. (2007). Carbon Nanotubes: Advanced Topics in the Synthesis, Structure, Properties and Applications.

[27] Ho K.C., Teow Y.H., Mohammad A.W., Ang W.L., Lee P.H. (2018). Development of graphene oxide (GO)/multi-walled carbon nanotubes (MWCNTs) nanocomposite conductive membranes for electrically enhanced fouling mitigation. J. Membr. Sci..

[28] Zhang X. (2008). Hydroentangling: A novel approach to high-speed fabrication of carbon nanotube membranes. Adv. Mater..

[29] Dumée L.F., Sears K., Schütz J., Finn N., Huynh C., Hawkins S., Duke M., Gray S. (2010). Characterization and evaluation of carbon nanotube Bucky-Paper membranes for direct contact membrane distillation. J. Membr. Sci..

[30] de Lannoy C.F., Jassby D., Davis D.D., Wiesner M.R. (2012). A highly electrically conductive polymer–multiwalled carbon nanotube nanocomposite membrane. J. Membr. Sci..

[31] de Lannoy C.-F., Soyer E., Wiesner M.R. (2013). Optimizing carbon nanotube-reinforced polysulfone ultrafiltration membranes through carboxylic acid functionalization. J. Membr. Sci..

[32] Dudchenko A.V., Rolf J., Russell K., Duan W., Jassby D. (2014). Organic fouling inhibition on electrically conducting carbon nanotube–polyvinyl alcohol composite ultrafiltration membranes. J. Membr. Sci..

[33] Liu W., Zhang X., Xu G., Bradford P.D., Wang X., Zhao H., Zhang Y., Jia Q., Yuan F.G., Li Q. (2011). Producing superior composites by winding carbon nanotubes onto a mandrel under a poly (vinyl alcohol) spray. Carbon.

[34] Zou J., Zhang X., Zhao J., Lei C., Zhao Y., Zhu Y., Li Q. (2016). Strengthening and toughening effects by strapping carbon nanotube cross-links with polymer molecules. Compos. Sci. Technol..

[35] Li S., Zhang X., Zhao J., Meng F., Xu G., Yong Z., Jia J., Zhang Z., Li Q. (2012). Enhancement of carbon nanotube fibres using different solvents and polymers. Compos. Sci. Technol..

[36] Zhou Y., Azumi R. (2016). Carbon nanotube based transparent conductive films: Progress, challenges, and perspectives. Sci. Technol. Adv. Mater..

[37] Song S., Xia S., Wei Y., Lv X., Sun S., Li Q. (2020). Fluoro-polymer-coated carbon nanotubes for improved interfacial interactions and dielectric properties in MWCNTs/PVDF composites. J. Mater. Sci..

[38] Yi G., Chen S., Quan X., Wei G., Fan X., Yu H. (2018). Enhanced separation performance of carbon nanotube–polyvinyl alcohol composite membranes for emulsified oily wastewater treatment under electrical assistance. Sep. Purif. Technol..

[39] Lalia B.S., Kochkodan V., Hashaikeh R., Hilal N. (2013). A review on membrane fabrication: Structure, properties and performance relationship. Desalination.

[40] Li J.-H., Shao X.-S., Zhou Q., Li M.-Z., Zhang Q.-Q. (2013). The double effects of silver nanoparticles on the PVDF membrane: Surface hydrophilicity and antifouling performance. Appl. Surf. Sci..

[41] Rathinavel S., Priyadharshini K., Panda D. (2021). A review on carbon nanotube: An overview of synthesis, properties, functionalization, characterization, and the application. Mater. Sci. Eng. B.

[42] Shen Y., Zhao W., Kang X., Xia H. (2010). A systematic insight into fouling propensity of soluble microbial products in membrane bioreactors based on hydrophobic interaction and size exclusion. J. Membr. Sci..

